# Attitudes, Education and Euthanasia

**DOI:** 10.1192/bjo.2025.10262

**Published:** 2025-06-20

**Authors:** Peter Carter, Matthew Lee

**Affiliations:** 1 North East London NHS Foundation Trust, London, United Kingdom; 2 Anglia Ruskin University, Chelmsford, United Kingdom

## Abstract

**Aims:** Following the recent passage of the Terminally Ill Adults (End of Life) Bill (2024) through UK Parliament, we set out to understand the views of psychiatrists towards assisted dying and the nature of their education in this area.

**Methods:** We selected 2 previous surveys. Using a pragmatic group, we developed a survey based on previously used questionnaires in this area. We gathered demographic data including religious affiliation, and country of primary medical qualification. We surveyed 44 psychiatrists in total.

**Results:** 27% had experience of undergraduate education on assisted dying euthanasia while 11% postgraduate education.

We adapted a survey form to measure attitudes towards euthanasia, giving a score out of 16. 31% scored 0 (with very positive attitudes to euthanasia), 11% scored 16 (very negative), and the median was 2. Participants with negative attitudes to euthanasia had a higher degree of religiosity, were more likely to train outside of the UK, but had similar age distribution to the general population.

The second part of the survey looked at euthanasia in psychiatric patients. Only 14% agreed that it should be allowed for psychiatric illnesses, with 27% unsure and 59% opposed.

The most acceptable statement (86% agreement) was that psychiatric patients can find themselves in a medically hopeless situation. 75% agreed that psychiatric patients could suffer unbearably. 70% agreed that psychiatric patients may run out of treatment options.

Participants opposed to euthanasia also opposed it in psychiatric patients. They were more likely to doubt the above statements, but only one participant disagreed with all statements. Most disagreed with the statement that “a death request can be well-regarded and considered not only as a symptom of illness” (66%).

Of participants in favour of euthanasia in general, 42% said they were unsure of legalising euthanasia for psychiatric patients, while 21% were opposed. This group was more uncertain of the statement that “euthanasia assessment can be compatible with psychotherapeutic relationship” (50% disagreed or unsure).

Most participants had little education in this area. International Medical Graduates and those self-described as religious were more likely to have negative perceptions of assisted dying in general. While there was agreement that suffering from mental illness could be unbearable, very few supported euthanasia for mental illness alone.

**Conclusion:** There is a notable lack of education on euthanasia at both undergraduate and postgraduate levels which is known to have a strong influence on attitudes to assisted dying. There are aspects in medical ethics and medical law which need to be incorporated into curricula for medical training.

